# Quantifying Methane and Methanol Metabolism of “*Methylotuvimicrobium buryatense*” 5GB1C under Substrate Limitation

**DOI:** 10.1128/mSystems.00748-19

**Published:** 2019-12-10

**Authors:** Lian He, Yanfen Fu, Mary E. Lidstrom

**Affiliations:** a Department of Chemical Engineering, University of Washington, Seattle, Washington, USA; b Department of Microbiology, University of Washington, Seattle, Washington, USA; California State University, Northridge

**Keywords:** ^13^C metabolic flux analysis, bioreactor, chemostat, isotopically nonstationary, type I methanotroph

## Abstract

Methanotrophic bacteria are a group of prokaryotes capable of using methane as their sole carbon and energy source. Although efforts have been made to simulate and elucidate their metabolism via computational approaches or ^13^C tracer analysis, major gaps still exist in our understanding of methanotrophic metabolism at the systems level. Particularly, direct measurements of system-wide fluxes are required to understand metabolic network function. Here, we quantified the central metabolic fluxes of a type I methanotroph, “*Methylotuvimicrobium buryatense*” 5GB1C, formerly Methylomicrobium buryatense 5GB1C, via ^13^C isotopically nonstationary metabolic flux analysis (INST-MFA). We performed labeling experiments on chemostat cultures by switching substrates from ^12^C to ^13^C input. Following the switch, we measured dynamic changes of labeling patterns and intracellular pool sizes of several intermediates, which were later used for data fitting and flux calculations. Through computational optimizations, we quantified methane and methanol metabolism at two growth rates (0.1 h^−1^ and 0.05 h^−1^). The resulting flux maps reveal a core consensus central metabolic flux phenotype across different growth conditions: a strong ribulose monophosphate cycle, a preference for the Embden-Meyerhof-Parnas pathway as the primary glycolytic pathway, and a tricarboxylic acid cycle showing small yet significant fluxes. This central metabolic consistency is further supported by a good linear correlation between fluxes at the two growth rates. Specific differences between methane and methanol growth observed previously are maintained under substrate limitation, albeit with smaller changes. The substrate oxidation and glycolysis pathways together contribute over 80% of total energy production, while other pathways play less important roles.

**IMPORTANCE** Methanotrophic metabolism has been under investigation for decades using biochemical and genetic approaches. Recently, a further step has been taken toward understanding methanotrophic metabolism in a quantitative manner by means of flux balance analysis (FBA), a mathematical approach that predicts fluxes constrained by mass balance and a few experimental measurements. However, no study has previously been undertaken to experimentally quantitate the complete methanotrophic central metabolism. The significance of this study is to fill such a gap by performing ^13^C INST-MFA on a fast-growing methanotroph. Our quantitative insights into the methanotrophic carbon and energy metabolism will pave the way for future FBA studies and set the stage for rational design of methanotrophic strains for industrial applications. Further, the experimental strategies can be applied to other methane or methanol utilizers, and the results will offer a unique and quantitative perspective of diverse methylotrophic metabolism.

## Abstract (precis)

Methanotrophic metabolism has been under investigation for decades using biochemical and genetic approaches. Recently, a further step has been taken toward understanding methanotrophic metabolism in a quantitative manner by means of flux balance analysis (FBA), a mathematical approach that predicts fluxes constrained by mass balance and a few experimental measurements. However, no study has previously been undertaken to experimentally quantitate the complete methanotrophic central metabolism. The significance of this study is to fill such a gap by performing ^13^C INST-MFA on a fast-growing methanotroph. Our quantitative insights into the methanotrophic carbon and energy metabolism will pave the way for future FBA studies and set the stage for rational design of methanotrophic strains for industrial applications. Further, the experimental strategies can be applied to other methane or methanol utilizers, and the results will offer a unique and quantitative perspective of diverse methylotrophic metabolism.

## INTRODUCTION

“*Methylotuvimicrobium buryatense*” 5GB1C, formerly Methylomicrobium buryatense 5GB1C (1), is a type I methanotroph employing the ribulose monophosphate (RuMP) cycle for carbon assimilation and growing only on one-carbon substrates (2). It has emerged as a promising candidate for industrial applications due to its fast growth, tolerance to high salinity and pH, and robust genetic tools (3, 4). Considerable fundamental and applied research has been carried out on this bacterium (5–11). An important question is how metabolic fluxes in central metabolism are organized when *M. buryatense* 5GB1C is grown on methane or methanol. Answering this question will help define central metabolic features in type I methanotrophs and their metabolic adaptations to environments supplied with different carbon sources. It may also allow us to identify pathways holding potential for industrial bioengineering.

Two main approaches have been used to assess the metabolic flux map in methanotrophs, flux balance analysis (FBA) and ^13^C metabolic flux analysis (MFA). FBA involves a genome-scale reconstruction model, which is subject to experimental constraints and mass balance based on reaction stoichiometry, and this approach has been applied to *M. buryatense* 5GB1C (12, 13). The model has predicted growth rates and yields in reasonable agreement with experimental measurements. Similar genome reconstruction models have been applied for other methanotrophs or methanol utilizers (14–16). However, such models do not directly measure fluxes, and even those constrained by experimental results contain significant uncertainties.

In order to directly measure fluxes, MFA is required. MFA employs ^13^C tracers to measure *in vivo* enzymatic reaction rates during isotopic and metabolic steady states (17–19). To this end, we have previously utilized metabolomics analysis and ^13^C labeling experiments under isotopic and metabolic steady states (13, 20), from which some key differences between methane and methanol metabolism were identified. For example, 2-keto-3-deoxy-6-phosphogluconate (KDPG), an intermediate in the Entner-Doudoroff (ED) pathway, exhibits a much higher pool size under growth on methanol than growth on methane, suggesting a possibly enhanced carbon flow through the ED pathway. In addition, the tricarboxylic acid (TCA) cycle operates oxidatively during growth on methane but likely branched during growth on methanol. However, it can be argued that the relative elevation of a metabolite pool does not always guarantee an increase in the carbon flow to its synthesis (21), providing uncertainty. Another limitation for labeling studies with one-carbon compounds is the complete labeling of most intermediates using steady-state methods. Only those intermediates downstream of carboxylation reactions can be assessed with steady-state analysis. In the case of *M. buryatense* 5GB1C, this limits information to the TCA cycle and related reactions. It also does not allow assessment of the operation of the methane or methanol oxidation pathway, the RuMP cycle, or the glycolysis pathway. Other techniques are required to examine the overall metabolism of *M. buryatense* 5GB1C quantitatively.

The alternative technique for measuring fluxes throughout central metabolism of *M. buryatense* 5GB1C is ^13^C isotopically nonstationary metabolic flux analysis (INST-MFA). During ^13^C INST-MFA, the carbon source is switched from a ^12^C substrate to its corresponding ^13^C isotopologue without perturbing the bacterial metabolism, and then the dynamic changes in labeling patterns of intracellular metabolites are measured. Since those changes are flux dependent, carbon fluxes can be calculated based on experimental measurements. ^13^C INST-MFA has been applied to cyanobacteria and plants feeding on CO_2_ as the sole carbon source (22–25), and it successfully captured their metabolic flux phenotypes in response to different growth conditions or genetic manipulations. However, it has not yet been used for any methanotroph growing on reduced one-carbon substrates. Because of many distinct physiological features between photoautotrophs and methanotrophs, the ^13^C labeling experiment for *M. buryatense* 5GB1C requires a redesign compared to these photoautotroph studies. Furthermore, as methane and methanol are a gas and a liquid, respectively, separate approaches have to be employed to deliver them into bacterial cultures and subsequently switch unlabeled carbons to labeled ones. In addition, methane is relatively insoluble in water, and addition of ^13^C-labeled methane into the headspace of tubes or vials may not generate an immediate usage by *M. buryatense* 5GB1C or a homogeneous distribution of the substrate in the culture. As a result, the metabolic steady-state assumption is unlikely to hold true.

In this work, we used ^13^C INST-MFA for analyzing both methane and methanol metabolism of *M. buryatense* 5GB1C. To address the above technical challenges and ensure an immediate switch to labeled substrates, we cultivated a continuous *M. buryatense* 5GB1C culture under substrate-limiting conditions. After determining flux distributions, we compared the features of methane and methanol metabolism of *M. buryatense* 5GB1C and then quantified the energy production and expenditure throughout the central metabolism. The metabolic flux phenotypes presented here will improve our understanding of methanotrophic metabolism and adaptation to various growth conditions. This information can also provide a knowledge basis for future metabolic engineering or multilevel omics studies.

## RESULTS

### ^13^C labeling experiment and physiological properties of *M. buryatense* 5GB1C.

**FIG 1:**
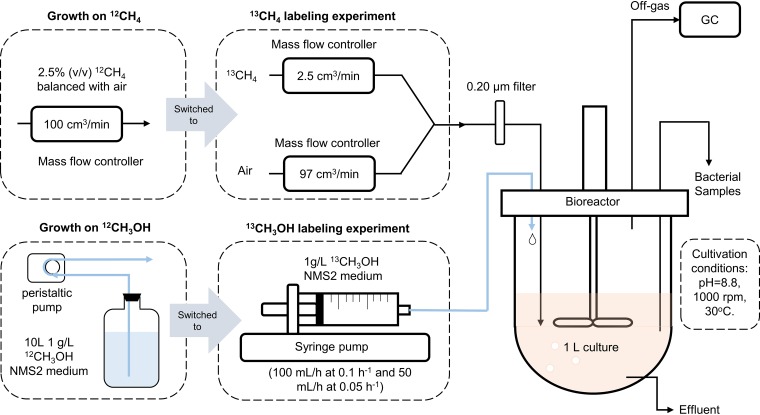
A schematic diagram of bioreactor setups for ^13^C labeling experiments. *M. buryatense* 5GB1C was grown on either 2.5% (vol/vol) CH_4_ balanced with air or 1 g/liter CH_3_OH in NMS2 medium. ^12^C substrates were later replaced by corresponding ^13^C substrates via different strategies. During growth on methanol, fresh ^12^CH_3_OH medium was delivered through a peristaltic pump from a medium carboy to the bioreactor. ^13^CH_3_OH medium was kept in a 60-ml syringe on a syringe pump. Upon the substrate switch, the peristaltic pump was stopped and the syringe pump was turned on immediately. For methane growth, 2.5% (vol/vol) CH_4_ was delivered to the bacterial cultures at 100 cm^3^/min. During the labeling experiment, this gas line was switched and connected to a gas mixture of ^13^CH_4_ and air. Their flow rates were 2.5 and 97 cm^3^/min, respectively, which were controlled by separate mass flow controllers. After the substrate switch, cell samples were collected at consecutive time points from 0 to 40 min, and labeling patterns of intracellular metabolites were then determined by LC-MS/MS.

*M. buryatense* 5GB1C cultures were grown under chemostat conditions in a bioreactor (see Fig. S1 in the supplemental material) (5, 6), and labeling experiments were performed under methane- and methanol-limiting conditions. We used 2.5% (vol/vol) CH_4_ gas balanced with air or 1 g/liter methanol as the feedstock. Two dilution rates, 0.1 h^−1^ and 0.05 h^−1^ (corresponding to doubling times of 6.9 h and 13.9 h, respectively), were tested to investigate whether metabolic fluxes at different growth rates would be correlated with each other. Under substrate-limiting conditions, methanol or methane concentration was low in the chemostat cultures, resulting in rapid ^12^C substrate deprivation in the medium after we switched to ^13^C substrate input. This ensures that the labeling pattern of the substrate does not vary temporally throughout the labeling experiment, making it possible to obtain a good fit to experimental data and a reliable flux calculation. As mentioned above, each substrate required a different strategy for switching substrates (Fig. 1). ^13^C-labeled methanol medium was delivered via a syringe pump. ^13^CH_4_ gas and air, with their flow rates controlled by separate mass flow controllers, were mixed within the gas delivery tubes before being supplied to *M. buryatense* 5GB1C cultures. Meanwhile, the bacterial culture was agitated vigorously at 1,000 rpm to ensure both an efficient gas-liquid transfer and a homogeneous culture.

10.1128/mSystems.00748-19.1 FIG S1 Bioreactor experiments. (a) ^13^CH_4_ labeling experiment. The above figure shows a typical ^13^CH_4_ labeling experiment, in which *M. buryatense* 5GB1 was grown in a bioreactor under chemostat conditions. The NMS2 medium contained no organic carbon substrates, and 2.5% (vol/vol) CH_4_ balanced with air was delivered into the bioreactor at a flow rate of 100 cm^3^/min. GC sampled off-gas every 15 min. We calculated the methane/O_2_ consumption rates and CO_2_ production rate (in mmol/h) based on the gas compositions between the inlet gas and off-gas. (b) ^13^CH_3_OH labeling experiment. The above figure shows a typical ^13^CH_3_OH labeling experiment. The NMS2 medium contained 1g/liter methanol, and air was delivered into the bioreactor at a flow rate of 100 cm^3^/min. The CO_2_ detection limit of the GC is ∼0.12% (vol/vol), below which the CO_2_ content would be measured as 0.00%. However, the zero percentage of CO_2_ displayed at the bottom figure does not mean that no CO_2_ was produced from *M. buryatense* 5GB1 cultures. Download FIG S1, TIF file, 1.4 MB. Copyright © 2019 He et al. 2019 He et al. This content is distributed under the terms of the Creative Commons Attribution 4.0 International license.

A set of physiological parameters (Table 1) was measured and used later to constrain our model. To maintain the same growth rate, *M. buryatense* 5GB1C consumes 30% to 40% more methane than methanol on a molar basis, partly because methane requires energy input to be oxidized to methanol. In addition, the formate production rate is over 7 times higher during growth on methanol than on methane. This trend is consistent with our previous reports for growth under substrate excess (5, 13). During growth on methane, OD_600_ is almost two times higher at the growth rate of 0.05 h^−1^ (4.6) than that at 0.1 h^−1^ (2.4), as expected for a gaseous substrate when the gas flow rate is kept constant. For the same amount of methane provided per unit time (100 cm^3^/min), the biomass dilution is half, so the steady-state OD_600_ should double. During growth on methanol, the OD_600_ (2.5) is independent of dilution rate, again as expected. In this case, the substrate is in the medium, such that increased dilution rate also increases the substrate input rate, and so the steady-state OD_600_ stays the same. For growth on both methane and methanol, the yield is similar at the two growth rates but different when comparing growth on methane to that on methanol. The O_2_/substrate ratio is an important parameter, as an indicator of the extent of oxidative phosphorylation. For growth on methane, one O_2_ is required for every methane utilized as part of the methane monooxygenase reaction, while any O_2_ utilized above that is used for oxidative phosphorylation. For growth on methanol, all of the O_2_ consumed is used for oxidative phosphorylation. The O_2_/methane ratio was about 1.1, while the O_2_/methanol ratio was about 0.6. The carbon conversion efficiency, which represents the percentage of carbons from the substrate that is converted to biomass, is higher for growth on methanol. These values suggest more CO_2_ production from methane metabolism. Finally, we show that both methane and methanol residues were extremely low (Table 1), which confirms substrate limitation for *M. buryatense* 5GB1C cultures.

**TABLE 1:** Physiological properties of *M. buryatense* 5GB1C

Substrate	Specific growth rate^*a*^ (h^−1^)	Substrate uptake rate (mmol/g/h)	Formate production rate (mmol/g/h)	OD_600_	Biomass yield (g/g)	CCE^*b*^ (%)	O_2_/CH_4_ or O_2_/CH_3_OH consumption ratio	Substrate residue^*c*^ (g/liter)
Methane	0.109 ± 0.003	9.02 ± 0.14	0.036 ± 0.002	2.42 ± 0.06	0.75 ± 0.01	48 ± 1	1.11 ± 0.00	∼1.2 × 10^−4^
Methane	0.054 ± 0.002	5.18 ± 0.10	0.005 ± 0.001	4.55 ± 0.19	0.65 ± 0.01	42 ± 1	1.14 ± 0.00	∼1.2 × 10^−4^
Methanol	0.098 ± 0.003	6.65 ± 0.16	0.28 ± 0.05	2.42 ± 0.18	0.42 ± 0.06	54 ± 8	0.60 ± 0.01	(8.7 ± 8.8) × 10^−4^
Methanol	0.053 ± 0.002	3.64 ± 0.13	0.19 ± 0.07	2.37 ± 0.23	0.43 ± 0.05	55 ± 7	0.56 ± 0.01	(5.8 ± 0.7) × 10^−4^

a Specific growth rates (or dilution rates) under chemostat conditions were determined as μ = amount of *M. buryatense* 5GB1C culture pumped out of the bioreactor per hour/total amount of bacterial culture in the bioreactor.

b Carbon conversion efficiency (CCE) is the percentage of carbon atoms from the substrate used for biomass synthesis.

c The methane residue in *M. buryatense* 5GB1C cultures was estimated as followed: methane residue = the volume fraction of methane in the gas phase (∼0.6% [vol/vol], measured by the GC) × total gas pressure (∼10^5^ Pa) × total mass of 1 liter *M. buryatense* 5GB1C culture (∼1 kg) × Henry’s law constant of methane (0.0012 mol/kg/10^5^ Pa) (42). The standard deviations are based on at least two biological replicates.

### ^13^C enrichments and pool sizes of central metabolites.

**FIG 2:**
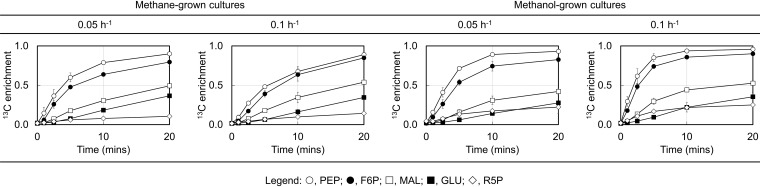
Dynamic changes of ^13^C enrichments of selected intermediates. ^13^C enrichments represent the percentage of ^13^C atoms in metabolites. Error bars are standard deviations from at least two biological replicates. Abbreviations: F6P, fructose 6-phosphate; GLU, glutamate; MAL, malate; PEP, phosphoenolpyruvate; R5P, ribose 5-phosphate.

^13^C INST-MFA was carried out by adding ^13^C-labeled substrates to steady-state substrate-limited cultures in the bioreactor. After the substrate switch, a time-series of culture samples were taken from 0 to 40 min. Figure 2 displays dynamic changes of ^13^C enrichments of selected intermediates. The labeling patterns of those intermediates are shown in Fig. S2, and all the mass isotopomer distribution (MID) data are detailed in Table S1. Overall, intermediates in the RuMP cycle and the Embden-Meyerhof-Parnas (EMP) pathway exhibit faster ^13^C incorporation than those in the TCA cycle and free amino acids, likely in part because the former pathways are proximal to the substrate oxidation pathway. One interesting observation is that the ^13^C fraction of ribose 5-phosphate (R5P) seems to stabilize between 10% and 20% after a 10-minute exposure to ^13^C substrates across all the growth conditions (Fig. 2). However, in cultures grown on ^13^CH_4_ for over five generations (approximately 15 h), R5P was fully labeled (20). One possible explanation for this phenomenon is that the R5P pool may be more heterogeneously distributed than other central metabolites, and thus, it takes a longer time for the labeled R5P in the RuMP cycle to be equilibrated with the R5P pool outside that pathway.

10.1128/mSystems.00748-19.2 FIG S2 Dynamic changes of mass isotopomer distributions of selected central metabolites. In each MID plot, Mn represents the fraction of different isotopologues, where *n* is the total ^13^C atoms in the isotopologue. Error bars are standard deviations from at least two biological replicates. Download FIG S2, PDF file, 1.1 MB. Copyright © 2019 He et al. 2019 He et al. This content is distributed under the terms of the Creative Commons Attribution 4.0 International license.

10.1128/mSystems.00748-19.5 TABLE S1 Mass isotopomer distributions. Download Table S1, XLSX file, 0.1 MB. Copyright © 2019 He et al. 2019 He et al. This content is distributed under the terms of the Creative Commons Attribution 4.0 International license.

**FIG 3:**
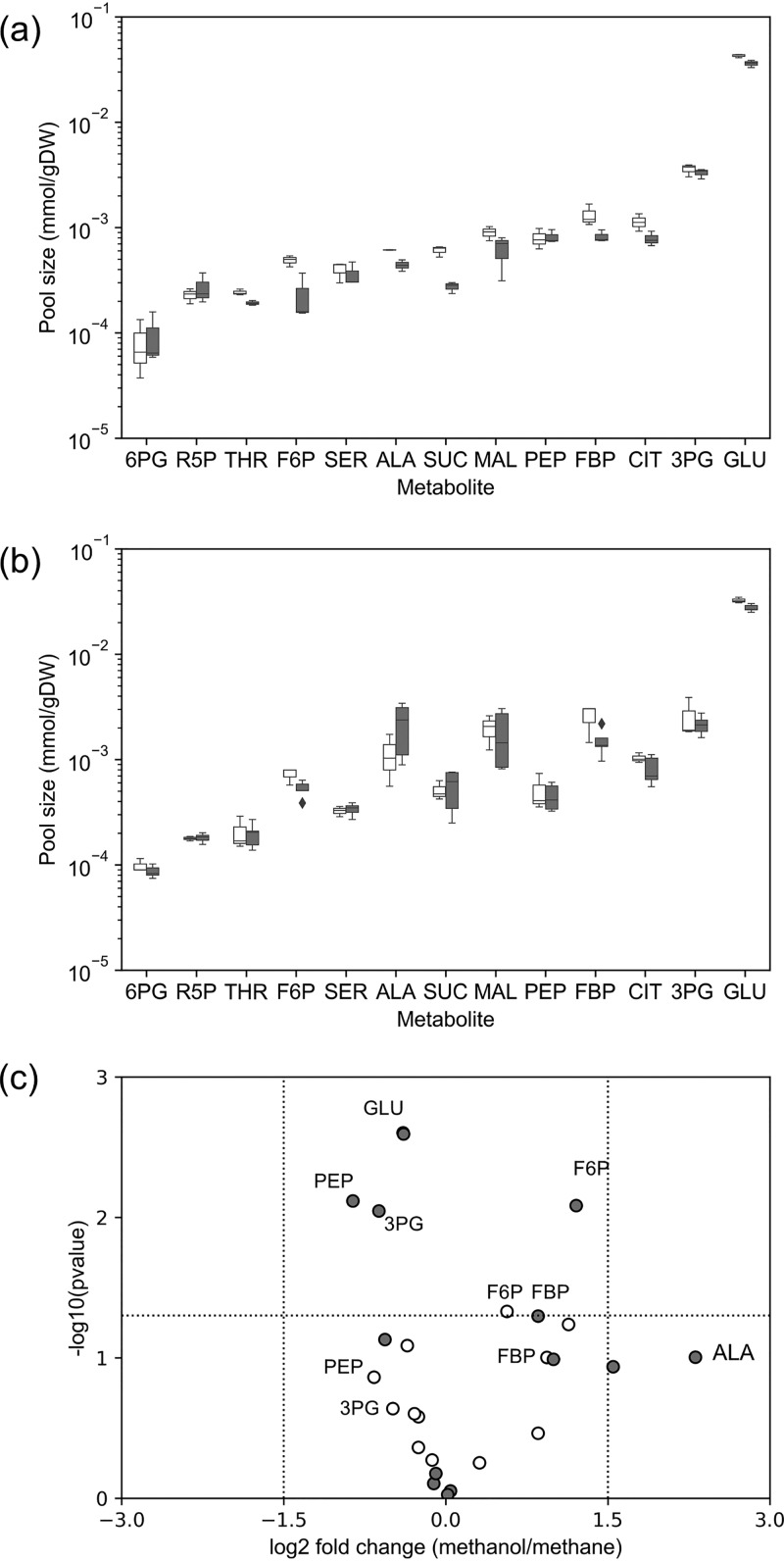
Pool sizes of central metabolites and free amino acids. (a) Metabolite pool sizes during growth on methane. (b) Metabolite pool sizes during growth on methanol. In panels a and b, the *y* axis represents pool size in milimoles per gram of dried cell weight (mmol/gDW) on a logarithmic scale. (c) Volcano plot of metabolite pool sizes. The fold changes were calculated as log_2_(pool sizes for methanol cultures/pool sizes for methane cultures). The *t* test was used to determine the *P* values. The horizontal dashed line shows the cutoff value (0.05). All white bars/markers represent results at 0.1 h^−1^, and gray bars/markers represent results at 0.05 h^−1^. Error bars are standard deviations from at least two biological replicates. Abbreviations: 3PG, 3-phosphoglycerate; 6PG, 6-phosphogluconate; ALA: alanine; CIT, citrate; F6P, fructose 6-phosphate; FBP, fructose 1,6-bisphosphate; GLU, glutamate; MAL, malate; PEP, phosphoenolpyruvate; R5P, ribose 5-phosphate; SER, serine; SUC, succinate; THR, threonine.

Metabolic pool sizes can affect ^13^C enrichment rates on short time scales. To obtain more precise flux calculations, we experimentally quantified the pool sizes of central metabolites by combining nonlabeled *M. buryatense* 5GB1C cultures with ^13^C-labeled Escherichia coli cultures. Intermediate pool sizes in E. coli were calibrated independently. We then estimated metabolite pool sizes of *M. buryatense* 5GB1C based on ^13^C/^12^C ratios of combined E. coli and *M. buryatense* 5GB1C samples (see Materials and Methods). The results show that pool sizes of intracellular metabolites in these substrate-limited cultures span from 10^−5^ to 10^−2 ^mmol/g dry weight (gDW) (Fig. 3a and b and Table S2). Glutamate shows the highest pool size, which also has one of the lowest ^13^C enrichment rates (Fig. 2). R5P and 6-phosphogluconate (6PG) are of much lower abundance than the rest of the metabolites. In general, metabolite pools at the two growth rates are similar. For the two different substrates, fructose 6-phosphate (F6P) and fructose 1,6-bisphosphate (FBP) show higher pool sizes during growth on methanol, while 3-phosphoglycerate (3PG) and phosphoenolpyruvate (PEP) have higher pool sizes during growth on methane (Fig. 3c). These results are in accordance with our previous findings (13), which indicate that some metabolic differences exist between methane and methanol metabolism. These differences can be observed as the log_2_ fold changes (Fig. 3c) of metabolite pools between methane and methanol growth.

10.1128/mSystems.00748-19.6 TABLE S2 Metabolite pool sizes. Download Table S2, XLSX file, 0.01 MB. Copyright © 2019 He et al. 2019 He et al. This content is distributed under the terms of the Creative Commons Attribution 4.0 International license.

### Flux distributions of *M. buryatense* 5GB1C during growth on methane or methanol.

**FIG 4:**
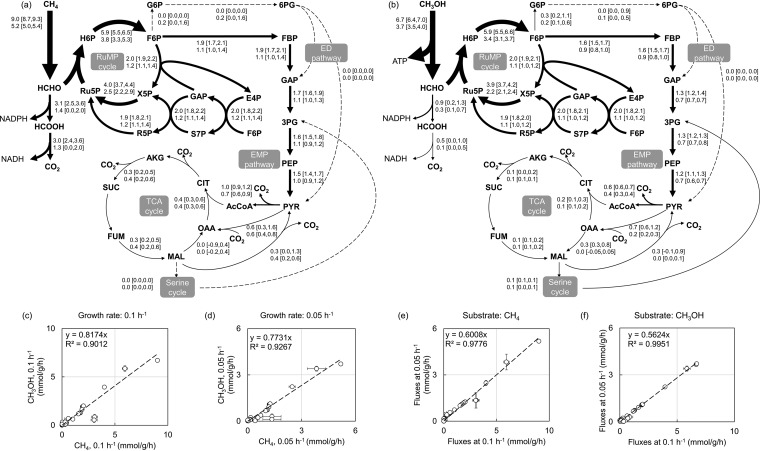
Metabolic flux phenotypes of *M. buryatense* 5GB1C under substrate limitation. (a) Flux map for growth on methane. (b) Flux map for growth on methanol. In the flux maps, upper numbers beside pathways are fluxes at 0.1 h^−1^ and lower numbers are fluxes at 0.05 h^−1^. Ninety-five percent confidence intervals are shown in brackets. The flux unit is milimoles per gram of dried cell weight per hour (mmol/g/h). SSR values under the four conditions are all statistically acceptable (Table S5). (c) Correlation of metabolic fluxes at 0.1 h^−1^ between methane and methanol metabolism. (d) Correlation of metabolic fluxes at 0.05 h^−1^ between methane and methanol metabolism. (e) Correlation of metabolic fluxes between the growth rates of 0.1 h^−1^ and 0.05 h^−1^ during growth on methane. (f) Correlation of metabolic fluxes between the growth rates of 0.1 h^−1^ and 0.05 h^−1^ during growth on methanol. In panels c to f, each open marker represents fluxes of a central metabolic reaction. Dashed lines are linear regression of *x* axis data against *y* axis data. Error bars represent standard deviations (SD) determined by the following equation: SD = (UB − LB)/3.92 (22), where UB and LB are 95% upper and lower bounds of confidence intervals, respectively. Abbreviations: 3PG, 3-phosphoglycerate; 6PG, 6-phosphogluconate; AcCoA, acetyl-CoA; AKG, alpha-ketoglutarate; CIT, citrate; E4P, erythrose 4-phosphate; F6P, fructose 6-phosphate; FBP, fructose 1,6-bisphosphate; FUM, fumarate; G6P, glucose 6-phosphate; GAP, glyceraldehyde 3-phosphate; MAL, malate; OAA, oxaloacetate; PEP, phosphoenolpyruvate; PYR, pyruvate; R5P, ribose 5-phosphate; Ru5P, ribulose 5-phopshate; S7P, sedoheptulose 7-phosphate; SUC, succinate; X5P, xylulose 5-phosphate.

To quantify the central metabolic fluxes of *M. buryatense* 5GB1C, MID data, pool sizes of central metabolites, and measured fluxes (i.e., substrate uptake rate, formate production rate, and specific growth rate) were fitted through computational optimizations. The resulting flux maps of *M. buryatense* 5GB1C are illustrated in Fig. 4a and b, where arrow thickness qualitatively represents flux strengths and numbers in brackets represent 95% confidence intervals. Central metabolic reactions are listed in Table S3, and detailed flux distribution results are shown in Table S4. MID data fitting results are presented in Fig. S3. Values for the sum of squared residuals (SSR) across all the growth conditions are all statistically accepted (Table S5).

10.1128/mSystems.00748-19.3 FIG S3 Data fitting results. Simulated MID data are plotted against corresponding measured MID data (Table S1). Each circle represents an MID data point. Download FIG S3, TIF file, 1.1 MB. Copyright © 2019 He et al. 2019 He et al. This content is distributed under the terms of the Creative Commons Attribution 4.0 International license.

10.1128/mSystems.00748-19.7 TABLE S3 Metabolic network and atom transitions. Download Table S3, XLSX file, 0.01 MB. Copyright © 2019 He et al. 2019 He et al. This content is distributed under the terms of the Creative Commons Attribution 4.0 International license.

10.1128/mSystems.00748-19.8 TABLE S4 Flux distributions. Download Table S4, XLSX file, 0.02 MB. Copyright © 2019 He et al. 2019 He et al. This content is distributed under the terms of the Creative Commons Attribution 4.0 International license.

10.1128/mSystems.00748-19.9 TABLE S5 Sum of squared residuals (SSR) across different growth conditions. Download Table S5, XLSX file, 0.01 MB. Copyright © 2019 He et al. 2019 He et al. This content is distributed under the terms of the Creative Commons Attribution 4.0 International license.

Overall, the patterns of methane and methanol metabolism are similar. The methane/methanol oxidation pathway and the RuMP cycle show the highest fluxes, consistent with higher metabolite ^13^C enrichment rates in those pathways. *M. buryatense* 5GB1C has two possible glycolytic pathways: the EMP pathway and the ED pathway. Based on our results, the EMP pathway is always the predominant glycolytic pathway under these growth conditions, while the ED pathway has a minimal flux. This result is also qualitatively supported by the observation that 3PG, an intermediate in the lower EMP pathway, has a much larger pool size (Fig. 3a and b) yet a higher ^13^C enrichment rate than 6PG (Fig. S4), which is the sole precursor to KDPG in the ED pathway. Further, when the phosphofructokinase reaction in the EMP pathway was set to zero flux *in silico*, the resulting SSR values were all increased by over three times (Table S5) and were no longer statistically acceptable. Collectively, these results suggest the EMP pathway is the principal glycolytic pathway in *M. buryatense* 5GB1C under substrate limitation.

10.1128/mSystems.00748-19.4 FIG S4 ^13^C enrichments of 3-phosphoglycerate (3PG) and 6-phosphogluconate (6PG) across different growth conditions. Black bars are ^13^C enrichments of 3PG, and white bars are ^13^C enrichments of 6PG. Error bars represent standard deviations from at least two biological replicates. Download FIG S4, TIF file, 1.0 MB. Copyright © 2019 He et al. 2019 He et al. This content is distributed under the terms of the Creative Commons Attribution 4.0 International license.

These results also suggest that fluxes through the TCA cycle are low, accounting for only 3 to 8% of substrate uptake rates. The best fits indicate that the TCA cycle is branched at the malate dehydrogenase reaction, and the remaining part operates in the oxidative direction. A large fraction of oxaloacetate (OAA) is replenished through the pyruvate carboxylase (PC) reaction. During growth on methanol, OAA seems to be exclusively produced from the PC reaction. Compared to the TCA cycle, a much lower carbon flux can be found in the serine cycle for both growth substrates (methanol, 0.1 ± 0.0 mmol/g/h; methane, 0.0 ± 0.0 mmol/g/h). The serine cycle serves as the major carbon assimilation pathway in type II methanotrophs. In contrast, in *M. buryatense* 5GB1C, a type I methanotroph, this cycle is incomplete, lacking reactions converting acetyl coenzyme A (CoA) to glyoxylate. It functions to convert two one-carbon units to acetyl-CoA, and it can also function as an alternate metabolic route for glycine and serine synthesis.

Major differences between methane and methanol metabolism can be found in the methane/methanol oxidation pathways. Table 1 shows that more methane is consumed than methanol at the same growth rate, while formate production is more active for growth on methanol. Further, the flux maps reveal that the carbon flow from methane or methanol bifurcates into the tetrahydromethanopterin (H_4_MPT) pathway (converting formaldehyde to formate) and the RuMP cycle in different proportions, suggesting a flexible metabolic branch point at the formaldehyde node. About 30% of the methane-derived formaldehyde is directly oxidized to CO_2_ via the H_4_MPT pathway and formate dehydrogenase (FDH) reaction, with the rest entering the RuMP cycle, while less than 8% methanol is directly converted to CO_2_ by this route during growth on methanol. Accordingly, even though the methanol uptake rate is lower than the methane uptake rate at the same growth rate (Table 1), a larger fraction of methanol enters the RuMP cycle. Inevitably, CO_2_ loss is more significant during growth on methane, which can be qualitatively validated by our gas chromatography (GC) measurements (Fig. S1). Specifically, the flux calculations show that about 50% methane and 20% methanol are eventually lost as CO_2_. However, we do not have any direct experimental measurement to constrain the overall CO_2_ production, and thus the FDH and the malic enzyme (ME) pathways sometimes show broader confidence intervals than other reactions.

Under these substrate-limited conditions, the majority of fluxes for methane and methanol metabolism show good linear correlations (*P* value < 0.01, Fig. 4c and d) at the two growth rates. The major outliers are the FDH and the H_4_MPT fluxes, as mentioned above. Moreover, for a single substrate, we observed nearly perfect linear correlations of flux distributions between the two dilution rates (*P* value < 0.01, Fig. 4e and f). These linear correlations indicate that for each substrate the relative flux distribution is relatively invariant and independent of the growth rate.

### Energy metabolism analysis of *M. buryatense* 5GB1C.

Based on the metabolic flux calculations, we examined the predicted energy production and expenditure in *M. buryatense* 5GB1C. This exercise assumed a direct coupling mode for the methane oxidation reaction, as suggested by a recent FBA study (12), and thus, we assumed that the electrons for methane oxidation directly come from the methanol dehydrogenase reaction. Under this scenario, the first two steps in the methane oxidation pathway generate no energy molecules (Table S3).

**FIG 5:**
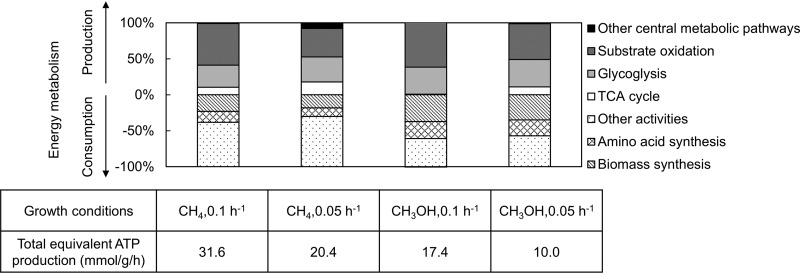
Energy metabolism analysis of *M. buryatense* 5GB1C. Total equivalent ATP production represents all the ATP produced from the central metabolism after each NAD(P)H is converted to 3 ATP. The stacked columns show relative energy contribution or consumption from major central metabolic pathways. Positive values represent energy production, and negative values represent energy consumption.

A detailed description of production and consumption of each energy molecule is given in Table S6. To obtain an estimation of relative energy contribution and consumption, we calculated the equivalent ATP contribution or consumption from different central metabolic pathways on the premise that one NAD(P)H equals 3 ATP, and then we normalized those values by the total equivalent ATP production from the central metabolism (Fig. 5). The results show that, compared to methanol growth conditions, more energy is produced from methane under substrate limitation. The substrate oxidation and the EMP pathways are two major energy suppliers, accounting for over 80% of the total energy production. The TCA cycle, on the other hand, plays a less important role, contributing less than 20% of the total energy production.

10.1128/mSystems.00748-19.10 TABLE S6 Quantification of energy production and expenditure in the central metabolism of *M. buryatense* 5GB1C (unit: mmol/g/h). Download Table S6, XLSX file, 0.01 MB. Copyright © 2019 He et al. 2019 He et al. This content is distributed under the terms of the Creative Commons Attribution 4.0 International license.

Notably, we found that the energy produced from the substrate oxidation pathway alone is enough for supporting biomass synthesis (Fig. 5), which consumes 20 to 40% of the total energy produced from central metabolism. Synthesis of amino acids also expends 10 to 20% energy. The above two activities together consume 30 to 60% of the total energy produced in *M. buryatense* 5GB1C. Compared to methane growth conditions, a greater fraction of energy is devoted to reproduction during growth on methanol; however, the absolute energy requirements are very similar for the two substrates at the same growth rate. Unlike a genome-scale reconstruction, our network includes reactions mostly in central metabolism, and thus, we cannot distinguish where *M. buryatense* 5GB1C uses the rest of the energy. For brevity, we combined all the energy surplus into the “other activities” category (Fig. 5), which includes energy expenditure for non-growth-associated ATP maintenance, active transport of nutrients from the environment, mobility, and other energy costs.

## DISCUSSION

In recent years, many efforts have been made to elucidate and quantitate the metabolism of methanotrophs that can use methane or methanol as the carbon and energy source. Most studies have relied on genome-scale reconstruction models, which have been successfully established as a common and convenient method for analyzing, simulating, and predicting cell metabolism across broad phylogenetic categories (26). However, even experimentally constrained FBA models have limitations and uncertainties. Moreover, for growth on methane or methanol, traditional ^13^C MFA under isotopic steady state offers limited insights into the core metabolism, since most metabolites are fully labeled with ^13^C atoms. To experimentally quantify metabolism of one-carbon substrates in RuMP cycle methanotrophs, ^13^C INST-MFA is currently the only viable approach. Here, we have employed ^13^C INST-MFA to quantitatively analyze methane and methanol metabolism of *M. buryatense* 5GB1C, an obligate type I methanotroph, growing under substrate-limiting conditions.

The resulting information is in agreement with ^13^C tracer labeling experiment results and FBA simulations in many aspects. First, the methane/methanol oxidation pathway exhibits the strongest fluxes, as predicted from FBA models (12, 13). Second, the EMP pathway proves to be the primary glycolytic pathway, while the ED pathway serves a supplementary role during growth on both methane and methanol. The same dominance of the EMP pathway has also been predicted in other type I methanotrophs, such as “*Methylotuvimicrobium alcaliphilum*” 20Z (27, 28) and *Methylomonas* sp. strain DH-1 (29). Moreover, this ^13^C INST-MFA study agrees with FBA studies (12) in showing that glycolytic flux accounts for only ∼20% (ranging from 17% to 27%) of the methane or methanol uptake rate. In comparison, a much larger flux is maintained in the RuMP cycle, primarily for driving carbon assimilation. Third, the TCA cycle flux is small but significant. Importantly, in spite of a weak flux, the TCA cycle is essential for *M. buryatense* 5GB1C metabolism, since interruption of the TCA cycle causes a severe growth defect (20). In comparison, the serine cycle is also weak, but it is not essential for *M. buryatense* 5GB1C, as interruption of the serine cycle via mutation does not inhibit growth (20). Finally, analysis of energy metabolism shows that the combination of the primary substrate oxidation pathways and the EMP pathway generates 80% of the energy and reducing power for cellular growth and metabolism.

Previous studies have been carried out with substrate sufficiency, while the work described here was carried out with substrate limitation, and we were able to identify a set of differences between these growth conditions. For example, with sufficient methanol, formate production accounts for about 10% of the total methanol consumption (5, 13), while this number drops to less than 5% under methanol-limiting conditions (Table 1). The same trend can be found in the serine cycle, which exhibits higher fluxes with excess methanol (13) than with limited methanol (Fig. 4b). Also, in the presence of sufficient carbon sources, we have observed more dramatic changes of metabolic pool sizes between methane and methanol metabolism (13). Finally, the increased flux through the ED pathway observed during growth at methanol sufficiency disappears during methanol-limited growth. All of the above comparisons suggest that substrate limitation, especially during growth on methanol, appears to diminish the scale of substrate-specific metabolic responses.

The relative constancy of metabolite pool sizes and flux distributions between methane and methanol metabolism under substrate limitations (Fig. 3 and 4) suggests that, despite varied substrates and growth rates, a “built-in” central metabolism is sustained in *M. buryatense* 5GB1C under substrate-limiting growth conditions. This feature suggests that the central metabolic fluxes of *M. buryatense* 5GB1C can be fine-tuned by manipulating its dilution rates, which could be a useful attribute for industrial application of this bacterium.

Another feature uncovered in this study is the flexibility of two nodes, formaldehyde and malate. Formaldehyde is partitioned between oxidation to formate and assimilation into the RuMP cycle. Our results show that depending on the substrate, this partitioning can change by over 3-fold, suggesting a flexible positioning of this branch point. Malate is involved in four reactions in *M. buryatense* 5GB1C, i.e., fumarase, malate dehydrogenase (MDH), malic enzyme (ME), and malate thiokinase (MTK) reactions. As shown in Fig. 4a and b, the fumarase reaction contributes to net malate synthesis under the growth conditions tested. Its deletion (specifically FumA) also results in a severe growth defect (20), suggesting its essentiality for *M. buryatense* 5GB1C metabolism. To maintain mass balance, malate produced from the fumarase reaction must be consumed via the other three pathways. As noted above, fluxes through the partial serine cycle are always low, and hence, the MTK reaction is low. The ME and MDH reactions are more flexible. The MDH reaction can operate in either direction according to the 95% confidence intervals. Furthermore, it has been confirmed that single knockout mutants of either MDH, ME, or serine glyoxylate aminotransferase (which is involved in the serine cycle) do not show any growth defect (6, 20). These results suggest that *M. buryatense* 5GB1C can spontaneously rewire carbon flows at the malate node without compromising biomass synthesis. Because of this flexibility, a futile cycle is possible: OAA → MAL → PYR + CO_2_ → OAA. This futile cycle consumes one ATP in total with no contribution to biomass synthesis. However, our best fit of flux distributions does not support significant activity of such a futile cycle, indicating that *M. buryatense* 5GB1C can avoid this energy trap. In summary, malate can function as a flexible metabolic branch point responding to environmental and genetic perturbations.

Interestingly, some metabolic flux features of *M. buryatense* 5GB1C can be also found in cyanobacteria using CO_2_ as the carbon source and light as the energy source. Prior ^13^C INST-MFA studies of several cyanobacterial species, such as *Synechocystis* sp. strain PCC 6803 (22), Synechococcus elongatus UTEX 2973 (30), and *Synechococcus* sp. strain PCC 7002 (25), have shown that they have a strong Calvin-Benson-Bassham cycle (which plays a similar role as the RuMP cycle in the type I methanotrophs) yet a weak TCA cycle. This resemblance is partly attributed to a similar architecture of the central metabolic network between type I methanotrophs and cyanobacteria, in which they both employ a metabolic cycle overlapped with the pentose phosphate pathway for consuming one-carbon substrates. Additionally, both can obtain adequate energy from either light or substrate oxidation/respiration, and thus, a strong TCA cycle is not vital. On the other hand, metabolites in *M. buryatense* 5GB1C exhibit generally higher ^13^C enrichments, or lower dilution factors estimated (Table S4), than wild-type cyanobacteria (22). One contributing factor for lower ^13^C enrichments in cyanobacteria could be the native metabolic channeling route located near carboxysomes, which confers metabolic efficiency but reduces cellular homogeneity. A recent study has shown that carboxysome-deficient mutants exhibit increased ^13^C enrichments in central metabolites (25). In the absence of such a microcompartment, metabolites are more evenly distributed in *M. buryatense* 5GB1C, and overall, they seem to be labeled sequentially from the RuMP cycle to downstream pathways (Fig. 2 and Fig. S2).

Recently, there has been an increasing interest in using methanotrophs to transform methane or methanol into value-added products (8–10, 31, 32). This study provides relevant information for such applications. For example, we have shown that the flux through the TCA cycle is weak in *M. buryatense* 5GB1C (Fig. 4a and b). Therefore, *M. buryatense* 5GB1C may not be naturally conducive to producing chemicals originating from the TCA cycle. It might be expected that engineering efforts will be required to enhance the carbon flow through the TCA cycle and/or the pool sizes of TCA cycle metabolites. Alternatively, because of a strong flux through the RuMP cycle, *M. buryatense* 5GB1C could be a suitable bacterial host for producing sugar-phosphate-derived products. The above suggestions can also be applied for some cyanobacterial species due to their similar central metabolic flux phenotype (30). Moreover, we have shown that methane metabolism generates more energy and reducing power than methanol (Fig. 5), especially NAD(P)H (Table S6). Accordingly, methane would be a better feedstock for methanotrophs if product biosynthesis requires a large quantity of reducing power, such as fatty-acid-derived chemical compounds (33).

### Conclusions.

In this study, we experimentally quantified the central metabolic fluxes in a type I methanotroph, *M. buryatense* 5GB1C, by means of ^13^C INST-MFA. This organism’s metabolic flux phenotype features a strong RuMP cycle, a preference for the EMP pathway over the ED pathway, and a weak TCA cycle. The methane/methanol oxidation pathway and the glycolysis pathway prove to be the major energy contributors in *M. buryatense* 5GB1C. Interestingly, our flux calculations suggest a largely stable central metabolism sustained under substrate limitation, which can be fine-tuned at several metabolic branch points, such as the malate and formaldehyde nodes, in response to changed growth conditions. Our quantitative insights into the type I methanotrophic metabolism can be used for the validation of future genome-scale model constructions and rational designs of methanotrophs.

## MATERIALS AND METHODS

### Bacterial cultivation and bioreactor setup.

*M. buryatense* 5GB1C was cultivated in NMS2 medium (3) at 30°C throughout this study. Single colonies of *M. buryatense* 5GB1C were inoculated into 250-ml serum vials containing 50 ml NMS2 medium. Either 50 ml methane or 2 g/liter methanol was supplied in vials. Seed cultures were grown for 1 or 2 days before inoculation into a bioreactor (New Brunswick BioFlo310, Eppendorf, CT, USA) containing 1 liter NMS2 medium. Bioreactor setups (Fig. 1) were as follows (5, 6): agitation speed was 1,000 rpm; pH was maintained at 8.8 by 2 mol/liter NaOH solution and monitored by a pH probe (Broadley-James Corporation, CA, USA); temperature was 30°C; and inlet gas flow rate was 100 cm^3^/min, which was regulated by a mass flow rate controller (Sierra Instruments, CA, USA). The inlet gas was sterilized by an autoclavable filter (0.22-μm Whatman Polydisc in-line filter; Aldrich, St. Louis, MO, USA) before being delivered to cultures. Off-gas composition was measured by a gas chromatograph (GC) (Shimadzu America, MD, USA), which sampled the off-gas every 15 min. An antifoam agent (Struktol J 660R; Struktol, Inc., Hamburg, Germany) was continuously applied into the bioreactor via a syringe pump (New Era 1000; New Era Pump Systems, Inc., NY, USA) at a rate of 20 μl/h. Under chemostat conditions, 10 liters NMS2 medium was prepared in a glass carboy and stirred at 500 rpm constantly. A peristaltic pump (Watson-Marlow Fluid Technology Group, MA, USA) was used to deliver fresh medium from the carboy into the bioreactor. Dilution rates, which were the same as the specific growth rates under chemostat conditions, were controlled by the feeding rate of fresh medium. The bioreactor, probes, tubes, and liquid medium were all autoclaved before use.

### ^13^C labeling experiments.

^13^C labeling experiments were performed under chemostat conditions. Under the methanol-limiting conditions, cultures were grown on 1 g/liter methanol at two dilution rates: 0.05 h^−1^ and 0.1 h^−1^. At time point zero, ^12^C medium was switched to ^13^C medium. The latter was kept in a 60-ml syringe and delivered to the bioreactor via the syringe pump. Following the substrate switch, cell samples were collected from 0 to 40 min. At each time point, ∼20-ml cell cultures were harvested on a Nylaflo filtration membrane (0.22 μm; Pall Life Sciences, NY, USA) through fast filtration. The membrane was immediately placed in sterile 50-ml tubes and quenched with liquid nitrogen. Under methane growth conditions, *M. buryatense* 5GB1C was supplied with 2.5% (vol/vol) methane gas balanced with air (Praxair, Inc., CT, USA). These labeling experiments were also performed at 0.1 h^−1^ and 0.05 h^−1^. Two separate mass flow controllers were used to maintain steady flows of ^13^CH_4_ gas (2.5 cm^3^/min) and air (97 cm^3^/min), which converged at a Y-shape connector. After ^13^CH_4_ and air gas tanks were opened, the flow rate of the mixture was first stabilized for 1 min before it was delivered to the bioreactor. This was carried out because the ^13^CH_4_ gas flow fluctuated in the beginning, which would perturb labeling patterns at the early time points. Cells were sampled at consecutive time points. For both methane and methanol growth, supernatant samples were taken from chemostat cultures before and after ^13^C labeling experiments, which were used for measuring formate and methanol concentrations. ^13^C-labeled substrates, ^13^CH_3_OH and ^13^CH_4_, were purchased from Sigma-Aldrich (purity ≥ 99%, Millipore Sigma, MO, USA).

### Measurement of formate production and methane/methanol consumption rates.

Methane or O_2_ consumption rates were calculated as follows:

(1) Vin⋅xN2,in=Vout⋅xN2,out

(2) $$\Delta V_{\text{O2}}=V_{\text{in}}\cdot x_{\text{O2},\text{in}}-V_{\text{out}}\cdot x_{\text{O2},\text{out}}$$

(3) $$\Delta V_{\text{CH4}}=V_{\text{in}}\cdot x_{\text{CH4},\text{in}}-V_{\text{out}}\cdot x_{\text{CH4},\text{out}}$$

where *V* represents the flow rate, subscripts “in” and “out” represent inlet and outlet gases, *x* represents volume fraction which was measured by the GC, and Δ*V* represents consumed gas volume per unit time. As described earlier, *V*_in_ was 100 cm^3^/min. Nitrogen gas was inert in our experiments, and thus, it can be used to correct the flow rate of other gas species (equations 1 to 3). The final gas substrate consumption rate was obtained by dividing Δ*V* by the molar volume (24.5 liters/mol at 25°C).

Methanol concentrations were measured by a methanol assay kit (BioVision, Inc., CA, USA). Formate concentrations in the supernatant were determined by an ICS-5000 ion chromatography system (Thermo Fisher Scientific, Waltham, MA) (13).

### Intracellular metabolite extraction and LC/MS-MS measurement.

Cell samples were first lyophilized overnight. Hot-water extraction procedures were applied for intracellular metabolite extraction (20, 34). Twenty milliliters boiling double-distilled water (ddH_2_O) was added into each sample, and then all samples were incubated in boiling water for 20 min. Samples were then cooled on ice for 30 min. Samples were then vortexed for ∼1 min, and the filter membranes were removed from the tubes. The resulting samples were centrifuged at 3,000 × *g* and 4°C for 30 min. Supernatants were collected, frozen by liquid nitrogen, and lyophilized. One milliliter sterile ddH_2_O was added to each dried sample, which was vortexed for ∼30 s and then centrifuged at 3,000 × *g* and 4°C. Supernatants were transferred to 1.5-ml microtubes, frozen, and lyophilized again. After overnight freeze-drying, 100 μl sterile ddH_2_O was added, and the samples were centrifuged at 20,000 × *g* for 30 min. The supernatant was collected and filtered using 0.22-μm Spin-X centrifuge tubes (Corning, Inc., NY, USA). The final samples were stored in 200-μl vials at −20°C before liquid chromatography-tandem mass spectrometry (LC-MS/MS) analysis.

Intracellular metabolites were separated by a SeQuant ZIC-pHILIC (5MYm polymer 150- by 2.1-mm polyether ether ketone [PEEK] coated) high-performance liquid chromatography (HPLC) column (EMD Millipore Corporation, Billerica, MA, USA), and labeling patterns were determined by a Waters Xevo mass spectrometer (Waters Corporation, Milford, MA, USA). Mobile phase A was 20 mM biocarbonate solution, and mobile phase B was acetonitrile. The flow rate was 0.15 ml/min. The initial gradient for mobile phase A was 15% for 0.5 min, and the gradient was increased to 85% in 20 min and held at 85% for 5 min. Then, the mobile A gradient was switched back to 15% for another 5.5 min. Multiple reaction monitors were the same as reported before (20).

### Metabolite pool size measurement.

To measure the metabolite pool size, E. coli intracellular metabolites were used as the internal standards. This method was modified from previous reports (35, 36). Single colonies of E. coli strain BL21(DE3) growing on LB agar medium were inoculated into M9 minimal medium supplied with 2 g/liter fully labeled glucose (U-^13^C_6_, purity ≥99%; Cambridge Isotope Laboratories, Inc., MA, USA). After overnight growth, seed cultures were inoculated into fresh ^13^C-labeled M9 medium at an initial OD_600_ of ∼0.02. About 4 h later, E. coli cultures (45 ml and OD_600_ of ≈0.32) were harvested on Nylaflo membranes through fast filtration, and these were placed in the same tubes together with nonlabeled *M. buryatense* 5GB1C samples obtained before ^13^C labeling experiments. Intracellular metabolites were extracted from the combined E. coli and *M. buryatense* 5GB1C samples, and their labeling patterns were analyzed by LC-MS/MS.

We calculated the intracellular metabolite pool size of each metabolite by the following equation:

(4) C13overall=(ME. coli×C13E. coli)+(M5G×C135G) ME. coli+M5G

where ^13^C_overall_ is the ^13^C enrichment of a metabolite from a combined sample; M_E. coli_ and M_5G_ represent the total amount of such metabolite in E. coli and *M. buryatense* 5GB1C samples, respectively; and ^13^C_E. coli_ and ^13^C_5G_ represent the ^13^C enrichment of this metabolite in E. coli and *M. buryatense* 5GB1C, respectively. The total amount of a central metabolite in E. coli or *M. buryatense* 5GB1C equals intracellular metabolite concentration (mmol/gDW) × OD_600_ × sample volume × conversion factor. The conversion factor is 0.20 gDW/OD/liter (5) for *M. buryatense* 5GB1C and 0.47 gDW/OD/liter (37, 38) for E. coli. To simply the calculation, we ignored the ^13^C contribution from *M. buryatense* 5GB1C cultures growing on ^12^CH_4_ or ^12^CH_3_OH, i.e., ^13^C_5G_ ≈ 0. Absolute intracellular pool sizes of the E. coli strain BL21(DE3) were calibrated independently. Specifically, in the first step of extraction, ^13^C-labeled E. coli was added with both boiling ddH_2_O and ^12^C standards of known concentrations. The rest of the procedures were the same as described earlier. All the chemicals were purchased from Millipore-Sigma (St. Louis, MO, USA).

### Metabolic network and flux calculations.

The metabolic network includes the methane or methanol oxidation pathway, the RuMP cycle, the EMP pathway, the ED pathway, the oxidative pentose phosphate (OPP) pathway, the TCA cycle, the anaplerotic pathway, and the partial serine cycle. Since copper was not limited in culture, particulate methane monooxygenase (pMMO) was the major enzyme responsible for methane oxidation into methanol (39), and thus, the soluble methane monooxygenase (sMMO) reaction was not considered in the model. Energy molecules, namely, ATP, NADH, and NADPH, were included in the reaction mixtures, but their mass balances were not strictly constrained. A complete list of reactions is shown in Table S3 in the supplemental material. INCA (40), a MATLAB toolbox, was used to calculate the *in vivo* flux distributions by minimizing the sum of squared residuals (SSR) between experimentally measured and computationally simulated variables, including labeling patterns, metabolite pool sizes, specific growth rates, and formate production rates. Calculations were carried out in MATLAB version R2017a (MathWorks, Inc, MA, USA). The minimum standard deviation for MID data was set to 0.01. At least 100 different initial guesses were tested to find the optimal fit. The chi-square test was used to examine if the SSR values were statistically acceptable.

The following assumptions were applied in the model. (i) For methanol growth, glycogen content accounted for 42% of total biomass, while this value was lower for growth on methane (5). (ii) The other biomass compositions were based on a previous report (12), and they were assumed to be the same for both methane and methanol growth (Table S3). (iii) Formate was the major by-product, and other by-products (e.g., acetate, lactate, and succinate) were excluded in the model due to their low quantities (5). (iv) Phosphoketolase pathways were excluded in the model, since their knockout mutants show unaltered phenotypes compared to wild-type strains (6). (v) Intracellular metabolites may not be homogeneously distributed in bacterial cells, which could result in metabolically inactive pools. To mimic this heterogeneity, we introduced dilution factors (23, 41) in the model, which represented fractions of such inactive metabolic pools.

### Data availability.

All the data are presented either in the main text or in the supplemental material.
