# Supplementary figures and images for: Quantifying Methane and Methanol Metabolism of “Methylotuvimicrobium buryatense” 5GB1C under Substrate Limitation
Source: mSystems. 2019 Dec 10;4(6):e00748-19. doi: 10.1128/mSystems.00748-19 (PMC6906744; doi:10.1128/mSystems.00748-19)

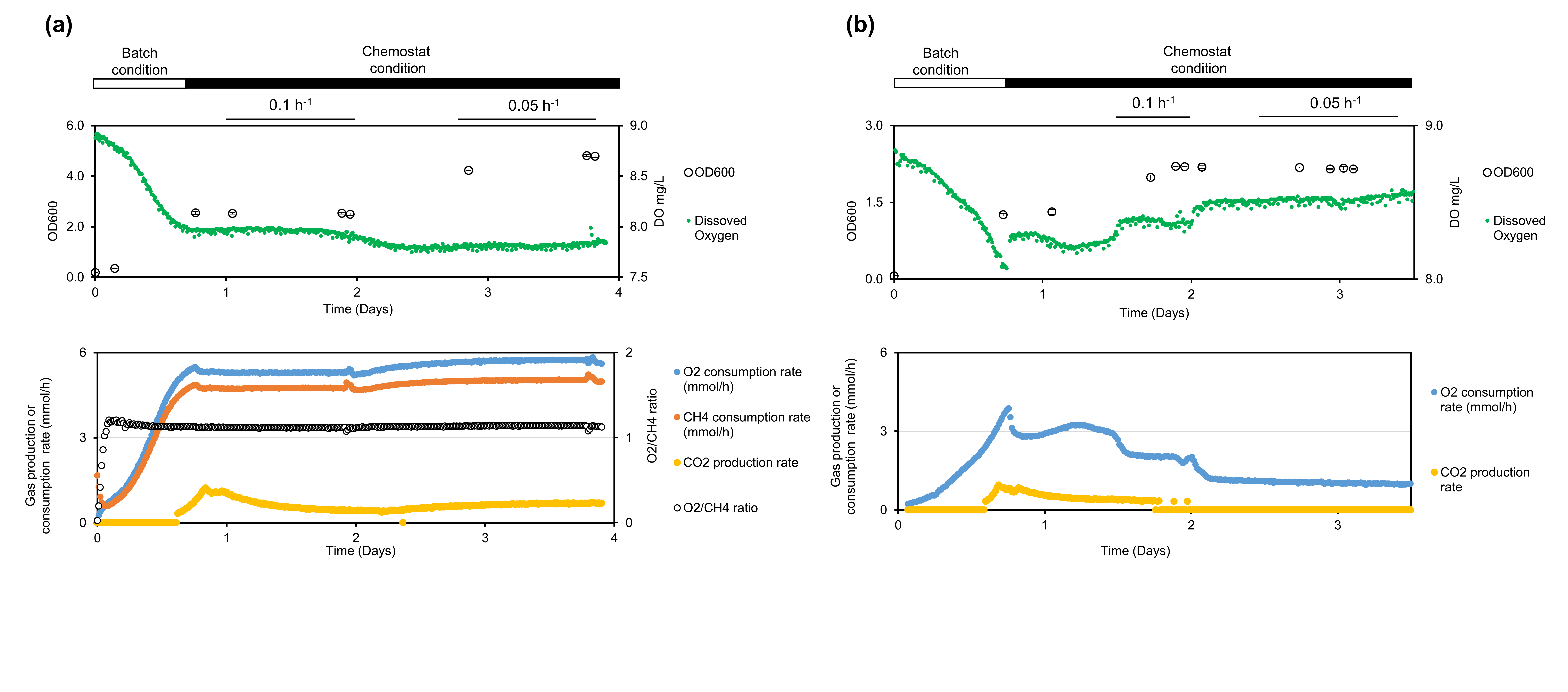

Supplement: FIG S1 [file mSystems.00748-19-sf001.tif]

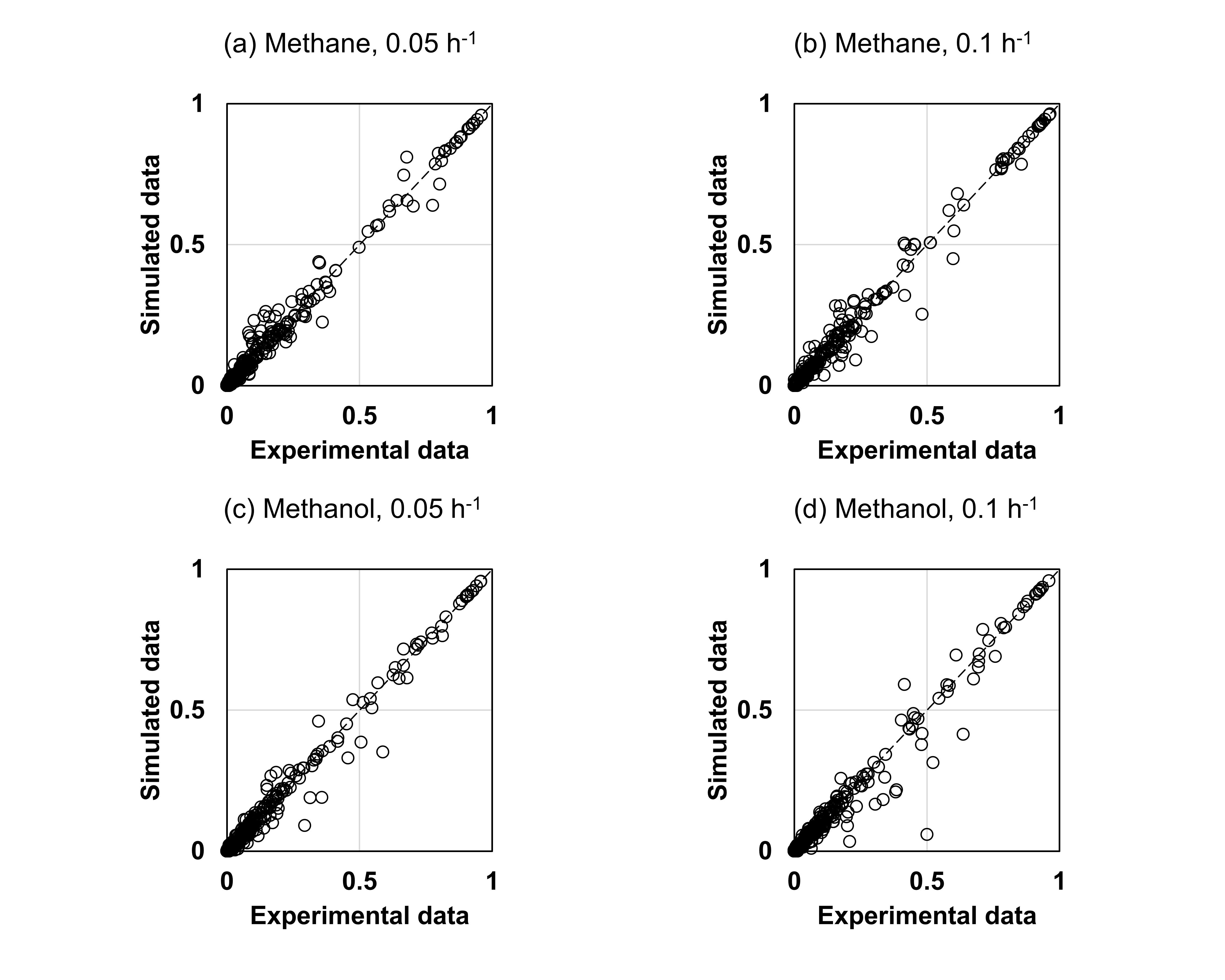

Supplement: FIG S3 [file mSystems.00748-19-sf003.tif]

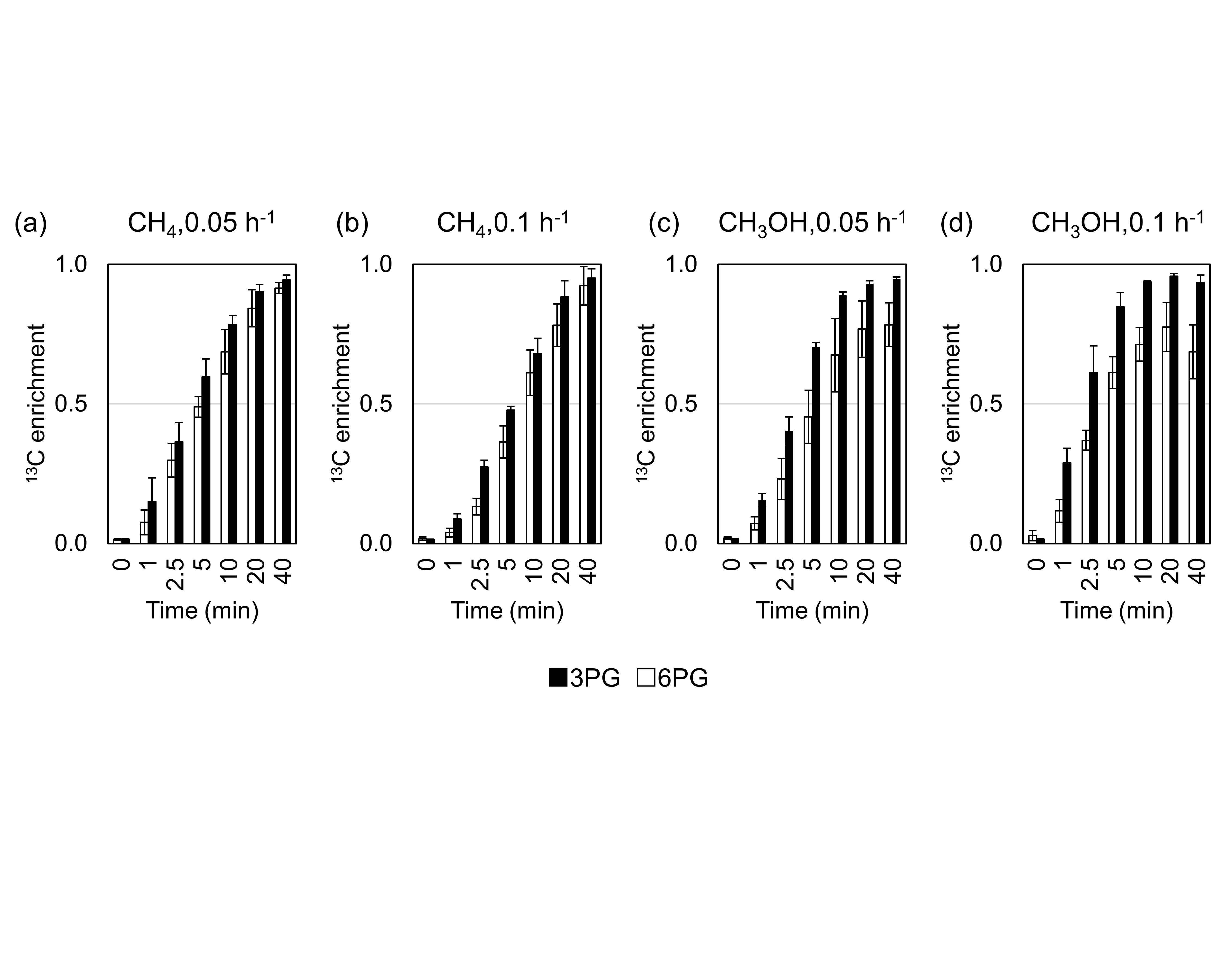

Supplement: FIG S4 [file mSystems.00748-19-sf004.tif]
