# Supplementary material for: Quantifying Methane and Methanol Metabolism of “Methylotuvimicrobium buryatense” 5GB1C under Substrate Limitation
Source: mSystems. 2019 Dec 10;4(6):e00748-19. doi: 10.1128/mSystems.00748-19 (PMC6906744; doi:10.1128/mSystems.00748-19)
Supplement: FIG S2 [file mSystems.00748-19-sf002.pdf]

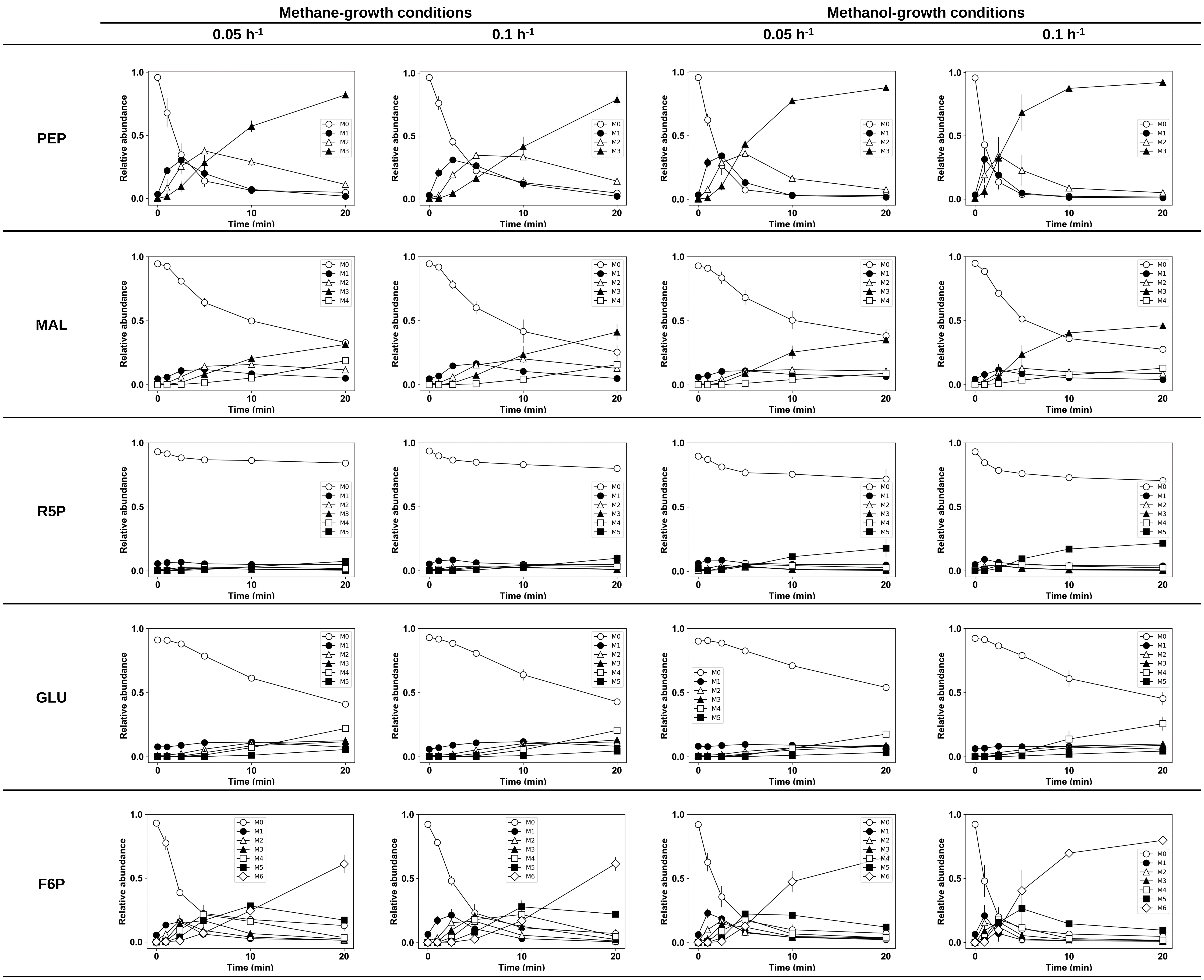

Methane-growth conditions
Methanol-growth conditions
0.05 h-1
0.1 h-1
0.05 h-1
0.1 h-1
PEP
MAL
R5P
GLU
F6P
Relative abundance
Time (min)
M0
M1
M2
M3
M4
M5
M6
